# Congenital diaphragmatic hernia presenting with symptoms within the first day of life; outcomes from a non-ECMO centre in Denmark

**DOI:** 10.1186/s12887-020-02072-2

**Published:** 2020-05-07

**Authors:** Ulla Lei Larsen, Søren Jepsen, Thomas Strøm, Niels Qvist, Palle Toft

**Affiliations:** 1 Research Unit for Department of Anaesthesiology & Intensive Care, Odense University Hospital, Odense, Denmark; University of Southern Denmark, Odense, Denmark; 2 OPEN, Odense Patient Data Explorative Network, Odense University Hospital/Institute of Clinical Research, University of Southern Denmark, Odense, Denmark; 3 Department of Anaesthesiology & Intensive Care, Odense University Hospital, Odense, Denmark; 4 Research Unit for Surgery, Odense University Hospital, Odense, Denmark: University of Southern Denmark, Odense, Denmark

**Keywords:** Infants, Congenital diaphragmatic hernia, Outcomes, Extra corporeal membrane oxygenation, Retrospective cohort study

## Abstract

**Background:**

Between 1998 and 2015, we report on the survival of congenital diaphragmatic hernia (CDH)-infants presenting with symptoms within the first 24 h of life, treated at Odense University Hospital (OUH), a tertiary referral non-extracorporeal membrane oxygenation (ECMO) hospital for paediatric surgery.

**Methods:**

We performed a retrospective cohort study of prospectively identified CDH-infants at our centre. Data from medical records and critical information systems were obtained. Baseline data included mode of delivery and infant condition. Outcome data included 24-h, 28-day, and 1 year mortality rates and management data included intensive care treatment, length of stay in the intensive care unit, time of discharge from hospital, and surgical intervention. Descriptive analyses were performed for all variables. Survivors and non-survivors were compared for baseline and treatment data.

**Results:**

Ninety-five infants were identified (44% female). Of these, 77% were left-sided hernias, 52% were diagnosed prenatally, and 6.4% had concurrent malformations. The 28-day mortality rate was 21.1%, and the 1 year mortality rate was 22.1%. Of the 21 non-survivors, nine died within the first 24 h, and 10 were sufficiently stabilised to undergo surgery. A statistically significant difference was observed between survivors and non-survivors regarding APGAR score at 1 and 5 min., prenatal diagnosis, body length at birth, and delivery at OUH.

**Conclusions:**

Our outcome results were comparable to published data from other centres, including centres using ECMO.

## Background

Congenital diaphragmatic hernia (CDH) is a rare, but serious congenital malformation. The reported overall mortality is between 40 and 48% depending on the CDH-population, and an incidence of 0.08–0.38/1000 live born infants is described [1, 2]. The majority of CDH-cases are left-sided, but right-sided and, in rare cases, bilateral hernias may also occur [3]. A wide range of associated malformations and syndromes have been described, with congenital heart malformations being the most frequent, with clear negative impacts on survival [3].

In some cases symptoms are absent or subtle, and these may be serendipitously diagnosed by coincidence. Late-presenting CDH has an overall good outcome [4], when compared with infants presenting with symptoms in the neonatal period, which often require stabilising intensive care therapy. Cardiopulmonary instability is the main challenge, as lung hypoplasia and vascular bed abnormalities cause pulmonary hypertension [5]. In severe cases, further deterioration increases right ventricular strain and eventually, circulatory failure may occur.

In recent decades, notable and improved survival of infants with CDH has generally been attributed to advances in cardiopulmonary resuscitation in the intensive care unit. These improvements have been related to the introduction of “lung-protective ventilation,” delayed surgery, and an increased focus on targeting pulmonary hypertension and circulatory stabilisation issues [6]. Extracorporeal membrane oxygenation (ECMO) is a well-established treatment modality for neonates with reversible circulatory or respiratory failure, with a well-documented impact on survival [7]. Many ECMO-centres offer treatment to CDH-infants, when conventional treatments fail. However, despite improved technology, ECMO treatment is associated with severe complications [8] and evidence of causal effect on long term survival in CDH-populations is lacking [9, 10].

The objective of this study was to report 24-h, 28-day, and 1 year mortality rates in infants with symptomatic congenital diaphragmatic hernia, treated at a tertiary non-ECMO centre in Denmark. In addition, we describe these infants in terms of pre- or postnatal diagnosis, referral or in-hospital born, management (surgical and intensive care treatment), demographics and clinical data. Finally, we compare collected variables between survivors and non-survivors.

## Methods

### Study design

We performed a retrospective cohort study of prospectively registered infants with symptomatic CDH.

### Ethical permission

The study was conducted after permission was obtained from the Danish Patient Safety Authority (No: 3–3013-1121/1), and the Danish data protection agency (No: 15/34128).

### The study group

The study focussed on a cohort of consecutive live-born CDH-cases from the western region of Denmark, born at Odense University Hospital (OUH) or referrals from peripheral hospitals in the region. Our centre is the only tertiary unit in the region treating CDH patients, and is one of two centres in Denmark. Thus, all children diagnosed with CDH from the western region of Denmark were treated at OUH. None were transferred for treatment elsewhere. The region has a population of approximately 3.2 million, covering more than half of the Danish population (The population of Denmark is 5.8 million, Danmarks statistik/2019).

### The study population

All infants treated at OUH were registered under the following diagnosis: Congenital Diaphragmatic Hernia (ICD-10 code: DQ790). Infants were registered prospectively, and all live-born infants were eligible for inclusion. We excluded infants presenting with symptoms 24 h after birth, thus defining symptomatic CDH as infants showing signs of life at birth, and presenting with symptoms within the first 24 h of life.

### The study period

A multidisciplinary CDH-infant management approach was implemented in 1997. The study period from 1998 to 2015 was chosen to reflect this organisational change.

Data were collected retrospectively from charts, medical notes, electronic journals, and critical information systems. Data were obtained for all infants with symptomatic CDH, treated at the intensive care unit at OUH from 1998 to 2015.

Mortality was recorded as: within the first 24 h of life, 1–28 days, and 29–365 days. The following baseline data were noted: gestational age, birth weight and body length at birth, sex, prenatal diagnosis, mode of delivery, APGAR scores at 1, 5, and 10 min., referral or in-hospital born, location of hernia and other malformations. Other variables included: postnatal management in the paediatric intensive care unit (PICU) (mode of ventilation, time on mechanical ventilation, vasopressor/inotrope treatment, sedation and pain management), surgical management, length of stay in PICU and length of stay in hospital.

### Postnatal management

Delivery of prenatally diagnosed infants was scheduled at our institution. Infants diagnosed postnatally at other hospitals were transported to our institution for further treatment. The management of CDH-infants at our hospital, initially implemented in 1997, included a strategy of early intubation and gentle ventilation. All infants needing mechanical ventilation were started on high-frequency oscillatory (HFO) ventilation (SensorMedics 3100A/B HFO Ventilator, Viasys Healthcare, USA). Further ventilation strategies and weaning were tailored to the individual clinical situation, and could also include conventional mechanical ventilation (CMV), continuous positive airway pressure (CPAP), or supplementary oxygen. All infants were sedated initially using continuous intravenous infusion or refractory morphine, fentanyl, and midazolam doses. Methadone, phenobarbital, and clonidine were preferred for weaning and the treatment of withdrawal symptoms. Infants were monitored by pre- and post-ductal saturation, continuous invasive measurements of blood pressure via an arterial line – umbilical preferred, and central venous access was also established. Our protocol also included the aggressive treatment of acidosis using sodium-bicarbonate. The target value for post-ductal saturation was > 95%. In severe cases with pulmonary hypertension, treatment with iNO (inhaled Nitric Oxide) was initiated by the intensivist in charge, and adequate circulation and perfusion were maintained with appropriate inotropes/vasopressors. Echocardiography and a plain chest x-ray were performed within the first 24 h of admission to PICU, and later when necessary.

Surgery was scheduled when infants were stable on minimal respiratory and circulatory support, without further episodes of pulmonary hypertensive crises (adhering to the “delayed surgery strategy” [11]). Enteral feeds were commenced from day one, and gradually increased up to the calculated basic need if tolerated by the infant. Parenteral nutrients were only supplied when enteral feeding was not adequate, after approximately 1 week. In all cases, surgery was performed using open abdominal access, and for large defects, a patch was inserted. The routine use of a chest tube after surgery was not practiced. Pleurocentesis was performed when a mediastinal shift (compromising respiratory or circulatory function) was observed due to excess filling of the intrapleural space with replacement fluid after surgery. The procedure was guided by chest x-ray, and in some cases ultrasound, to minimise the risk of further complications.

Changes in management over the study period were noted; treatment with surfactants became more restricted as no benefit had been shown in mature CDH infants (administered only for premature cases) [12], and enteral administrated Sildenafil was introduced in the treatment of more severe cases presenting with pulmonary hypertension and refractory to iNO-treatment. Sildenafil was continued after discharge and the paediatric cardiologist team conducted weaning of the drug thereafter. Adequate circulation/perfusion was maintained using inotrope/vasopressor therapy. Dopamine and nor-epinephrine were first-line choices, but during the study period, dobutamine was more often replaced by milrinone, as a second-line treatment in cases with severe pulmonary hypertension. In some severe cases epinephrine was also administrated.

Our institution provides ECMO-treatment for adults with cardiac failure. Treatment of infants > 2 kg can be initiated and thereafter transferred to a paediatric ECMO-centre. None of the study cases were treated with ECMO, either at our institution or elsewhere.

### Statistical analyses

Mortality was recorded as follows: before 24 h, 1–28 days, and 29–365 days. Descriptive analyses were performed on all cases; survivors and non-survivors. Baseline data were presented as median values, or as percentages. Continuous non-parametric data were summarised as median and interquartile range values (25th and 75th percentile), and categorical data were summarised as percentages. Groups of survivors and non-survivors were compared using the Wilcoxon rank-sum test for continuous data, and the Chi-square test for categorical data.

Treatment and management of the cases during PICU-stay was presented as a percentage, or a median value (time), with interquartile ranges (25th and 75th percentile). All analyses were performed using STATA/IC15.0 (Stata Statistical Software: Release 15. College Station, TX, USA: StataCorp LLC). *P*-values < 0.05 were considered statistically significant.

## Results

**Fig. 1:**
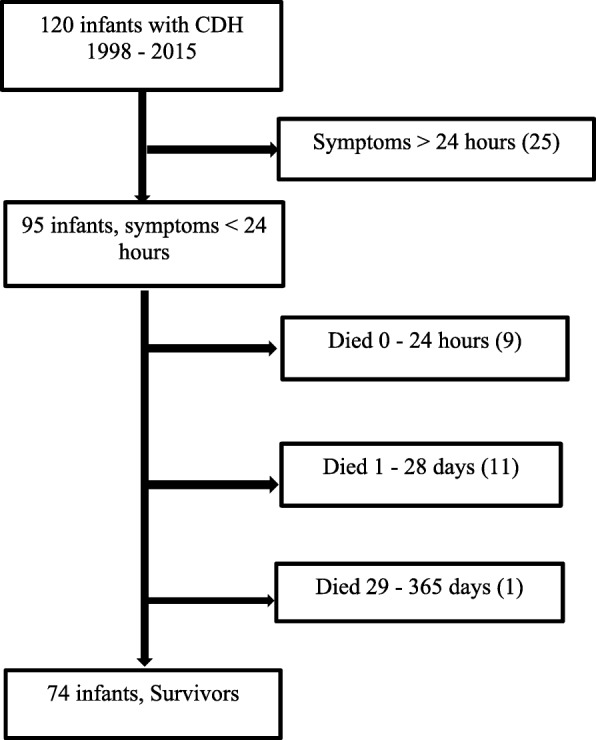
Flowchart of cases included in the study population

We identified 120 patients with CDH; 95 presented with symptoms during the first 24 h and were included in the study population. Twenty-five infants presented with symptoms after 24 h of life and these cases were excluded from the study. The flowchart is shown in Fig. 1.

Nine infants died during the first 24 h (9.5%), 11 infants died at 1–28 days (11.6%), and one infant died after day 28 (1.1%). In total 21 died < 1 year (Table 1). The excluded infants with late-onset symptoms (later than 24 h) all survived.

**Table 1:** Mortality and time of death for symptomatic CDH non-survivors

Mortality	Symptomatic CDH
28-day mortality	21.1%
1-year mortality	22.1%
Death before 24 h	9/95
Death before 48 h	11/95
Death before surgery	11/95
Death before PICU discharge	20/95
Death before hospital discharge	21/95
Death < 28 day	20/95
Death < 1 year	21/95

The one death noted after day 28, represents an infant born prematurely at the gestational age of 30 weeks, with a birth weight of 1.1 kg, and presenting with a left-sided hernia. No other malformations were noted, and initial surgical repair was performed with patch repair. The infant was successfully discharged from PICU after 29 days, although re-admitted shortly for surgery due to hernia recurrence. The infant was transferred back to the paediatric department of the local hospital, but died (unknown event) before home discharge.

Baseline data are shown (Table 2). Baseline data from one infant was missing, and APGAR scores were not available for three infants.

**Table 2:** Baseline data on our CDH-population

Baseline data (N)	All CDH cases	Survivors (74)	Non-survivors (21)	***P***-value
Sex (95), male	53 (55.8%)	40 (54.1%)	13 (61.9%)	NS
Gestational age (94)	38.5 (36.6–40)	38.6 (36.7–40)	38.3 (36.1–39.8)	NS
Birth weight, g (94)	3105 (2700–3550)	3150 (2700–3550)	3000 (2200–3350)	NS
Birth length, cm (94)	50 (48–52)	50 (49–52)	48 (44–50)	0.002
Prenatal diagnosis (94)	49 (52.1%)	33 (44.6%)	16 (76.2%)	0.017
In-born, OUH (94)	50 (53.2%)	34 (45.9%)	16 (76.2%)	0.012
Caesarean Section (94)	32 (34%)	25 (33.8%)	7 (33.3%)	NS
Associated malformations (94)	6 (6.4%)	4 (4.3%)	2 (9.5%)	NS
Hernia location (94)				
Left	71 (74.7%)	58	13	NS
Right	20 (21.1%)	13	7	
Bilateral	1 (1.1%)	1	0	
Not available	3 (3.2%)	2	1	
APGAR 1 min (91)	7 (4–9)	8 (6–9)	4 (2–6)	0.000
APGAR 5 min (80)	7 (6–9)	8 (6–9)	5.5 (4.5–7)	0.002
APGAR 10 min (39)	8 (7–10)	8 (7–10)	7 (5–9)	NS

Comparisons between survivors and non-survivors. Data are presented as percentages or median values and interquartile ranges (25th–75th percentile). Groups of survivors and non-survivors were compared using the Wilcoxon rank-sum test for continuous data, and the Chi-square test for categorical data. NS: Non-significant

Baseline data were compared between survivors and non-survivors. We observed non-survivors were more frequently diagnosed prenatally than survivors (*P* value 0,017) also, birth length was significantly different; non-survivors were shorter than survivors (*P* value 0,002). However, birth weight, sex, gestational age, indication for caesarean section, associated malformations, and hernia location did not show any significant differences between groups.

The majority of the study population (77.2%) presented with left-sided hernias (71/92 – three were undocumented). A right-sided hernia was present in 7/21 (33.3%) non-survivors, and 13/74 (17.6%) survivors, but this observation was not statistically significant. Overall, a right-sided hernia was noted in 20/92 (21.7%) infants. One infant had a bilateral hernia and survived.

In our cohort, 25 infants (27.5%) had an APGAR score at 1 min between 0 and 4, 26 infants (28.6%) had a score between 5 and 7, and 40 infants (44.0%) scored > 7. APGAR scores at both 1 and 5 min were significantly lower for non-survivors.

For 38 infants, all three APGAR scores (1 min, 5 min, 10 min) were available. APGAR scores at 10 min were only available in 39/95 infants and of these, seven were non-survivors (median = 7). For APGAR scores at 10 min, no significant differences were noted between survivors and non-survivors. For infants with APGAR score at 1 min < 7, 59% had missing APGAR data at 10 min. and for those with APGAR score at 1 min > 7, 48% had missing data. For infants with APGAR scores at 1 and 5 min > 9, 44% had missing data at 10 min.

Of the non-survivors, 10 (10/21) were initially sufficiently stabilised to undergo surgery, with five (50%) requiring patch repair when compared to survivors, where 12 (16%) needed patch repair. Overall hernia recurrence was noted in eight cases, where five (62.5%) initially needed patch repair.

Associated malformations occurred in six cases, of which two were non-survivors. The most frequent malformation was oesophageal atresia, with and without fistula. Also, chromosomal anomalies, cardiovascular, and minor urogenital malformations were observed. PICU management and treatment regimens are shown (Table 3). Not unexpectedly, we observed more advanced treatments in the non-survivor group, as all infants required mechanical ventilation, vasopressor/inotropic support and sedation. Stay durations on the group of survivors are also reported (Table 4).

**Table 3:** Intensive care and surgical management during the study period

Management/Treatment	All CDH cases	Survivors	Non-survivors
Mechanical ventilation	92 (97%)	71 (96%)	21 (100%)
HFO	75 (79%)	56 (76%)	19 (90%)
iNO	36 (38%)	18 (24%)	18 (86%)
Magnesium, iv	14 (15%)	6 (8%)	8 (38%)
Sildenafil, ga	13 (14%)	6 (8%)	7 (33%)
Surfactant	13 (14%)	5 (7%)	8 (38%)
Vasoactive drugs	60 (63%)	39 (53%)	21 (100%)
Nor-epinephrine	15 (16%)	6 (8%)	9 (43%)
Dopamine	51 (54%)	34 (46%)	17 (81%)
Dobutamine	16 (17%)	9 (12%)	7 (33%)
Milrinone	14 (15%)	5 (7%)	9 (43%)
Epinephrine	12 (13%)	1 (1%)	11 (52%)
Sedatives	80 (84%)	59 (80%)	21 (100%)
Fentanyl	77 (81%)	57 (77%)	20 (95%)
Midazolam	54 (57%)	39 (53%)	18 (86%)
Methadone	11 (12%)	11 (15%)	0 (0%)
Phenobarbital	43 (45%)	37 (50%)	6 (29%)
Clonidine	10 (11%)	9 (12%)	1 (5%)
Operation	84 (88%)	74 (100%)	10 (48%)
Patch repair (operation)	17 (20%)	12 (16%)	5 (50%)
Recurrent hernia (operation)	8 (8%)	7 (10%)	1 (1%)

Data are presented as percentages. *iv* intravenous, *ga* gastrointestinal

**Table 4:** Stay duration for CDH-survivors

Survivors, ***N*** = 73	days
Time on mechanical ventilation	6.4 (2.9–16.4)
LOS-PICU	8.3 (4.4–18.6)
LOS- Hospital	26.1 (15.9–52.8)

Time on mechanical ventilation, number of days in intensive care unit, (length of stay, LOS-PICU), and the total number of days in hospital (length of stay, LOS-hospital) for CDH-survivors. Data from one infant is missing. Data are represented as the median and interquartile ranges (25th – 75th percentile)

Placing a chest tube was not a routine procedure; however pleurocentesis was performed if clinically indicated. Unfortunately, the procedure pleurocentesis was not included in our study protocol and therefore this data was not retrieved in a structured manner.

## Discussion

We observed that survival in our cohort compared favourably with reports from other centres. Our data included all cases of symptomatic CDH admitted during the study period; this included cases with factors believed to negatively impact on survival, e.g. low birth weight [13], prematurity [13], right-sided hernia [14], prenatal diagnosis [13], and associated malformations [15]. Other risk factors associated with mortality, i.e. liver-up [16] and the lung-to-head ratio [16] were not assessed.

Of note, the CDH cases presenting with symptoms after 24 h of life (and excluded from this study) were admitted to the intensive care unit for postoperative care at a median age of 340.9 days (147.04–785 days). Including all CDH cases at our hospital during the study period, both symptomatic and late-presentations (symptoms after 24 h of life); we note an overall mortality of 17.5%.

Comparisons of APGAR scores 1 + 5 min, showed significantly lower values for non-survivors, which correlated well with previously published data [17]. APGAR scores at 10 min did not show the same trend. As infants with low scores at 1 min were more likely to have missing values at 10 min, we speculated that more severely affected infants were already undergoing supportive treatments within 10 min after birth, including sedation, making an APGAR score non-applicable. Also, infants with high APGAR scores at 1 and 5 min had a high percentage of missing values. We concluded that APGAR scores at 10 min were not uniformly collected during the study period, and therefore should not be taken into account as a predictor of outcome in our CDH-population.

We used HFO ventilation as the first-line mode of respiratory support. Since data collection, the VICI-trail; a multicentre randomised study on primary ventilation mode (CMV vs. HFO) was published [18]. The study found no significant differences in primary outcomes (death or bronchopulmonary dysplasia), but reported a benefit of CMV to secondary outcomes. The majority of centres had access to ECMO, but no differences in outcomes were found between EMCO- and non-ECMO centres. A study limitation was a slow inclusion rate; therefore it was terminated early before the calculated sample size was reached [18].

Our centre is one of two in Denmark caring for infants with CDH. We provide advanced intensive care for neonates and ECMO is currently not offered to CDH patients. However, our centre treats adult patients, and when indicated for other diagnosis, infant ECMO treatment (minimum weight; 2 kg) can be initiated (by our local team or by an ECMO-transport team) and thereafter transferred to a paediatric ECMO-centre.

Many established ECMO-centres provide treatment for CDH-infants, when conventional therapy fails. As very few randomised trails evaluating ECMO treatment include CDH-patients, the indication of impact on survival is primarily based on case and retrospective cohort studies [9, 19]. Also, comparing outcome between centres can be challenging due to differences in patient selection, variations in indications and cut-off values for initiating ECMO treatment [9, 20].

ECMO centres have increased their CDH-survival after implementing or optimising ECMO-protocols and mortality rates ranging from 5 to 24% have been reported [20–23]. Alongside ECMO-treatment, other modalities targeting pulmonary hypertension and lung protection have been implemented or refined over recent decades [24]. Thus, centres without access to ECMO, also report increased survival rates correlating with the introduction of multidisciplinary and more aggressive multimodal treatment approaches. As described at our hospital, organisational and management changes resulted in significant improvements in outcomes for our CDH population, reducing mortality from 67 to 23% [25]. Other centres have reported mortality rates between 13 and 34% [26–28]. In recently published guidelines, the CDH-EURO consortium (2015) stated that the benefits of ECMO for CDH treatment remained unclear, and provided grade D recommendations for initiating treatment. However, the following criteria were stated: preductal saturation < 85% or postductal saturation < 70%, respiratory acidosis with a pH < 7.15, peak inspiratory pressure > 28 cm H_2_O, or mean airway pressure > 17 cm H_2_O, metabolic acidosis with lactate ≥5 mmol/l and pH < 7.15, shock refractory to treatment and with urine output < 0.5 ml/kg/h for at least 12–24 h, and oxygenation index (OI) ≥ 40 present for at least three hours [29]. These recommendations were consistent with the guidelines published in 2010 [30], and are marginally more conservative than those put forward by The Extracorporeal Life Support Organization (ELSO; www.elso.org).

The true impact of ECMO treatment for CDH management is still not fully elucidated. The published data often represents different populations and treatment approaches, making direct comparisons challenging. Furthermore, studies addressing causal effects are lacking for CDH populations.

Our study had several limitations. Despite adhering to the same management protocol throughout the study period, adjustments and minor changes were made according to justified best clinical practise [30]. Prenatal care improved as first- and second-trimester ultrasound monitoring was introduced as a routine procedure during pregnancy, thereby influencing the frequency of prenatally diagnosed cases. Prenatal diagnostics increased throughout the first, second, and third part of the study period; i.e. 19.4, 59.4 and 78.1%, respectively. The survival rates for these periods were 77.4, 81.3, and 75.0%, respectively. From 2016 to 2019, the prenatal detection rate was 83.3%, and the survival rate was 83.3% (unpublished data).

As we reported from a single centre (not an epidemiological study), our data may have been subjected to selection bias, i.e. the small number of associated malformations at birth (6/94, 6.4%). The number of cases with associated malformations was less than expected, as other population-based/epidemiological studies reported concurrent malformations in approximately 32% live-born CDH cases [2]. We speculate this low frequency may have been due to counselling, either at local hospitals or our centre, resulting in elective terminations if other malformations were present.

Another limitation was the lack of parameters evaluating the degree of pulmonary hypoplasia. We reported on several indicators of poor outcomes, but not specifically the degree of pulmonary hypoplasia. This factor is a significant contributor, alongside persistent pulmonary hypertension, to CDH outcomes and is a main feature of CDH [31].

Lung-to-head ratio evaluates lung volume prenatally in CDH infants, and is used as a prognostic marker for outcome [16]. Magnetic resonance imaging is also used to prenatally evaluate the degree of lung hypoplasia, however, this modality has only recently been taken up at our institution and was therefore not evaluated here [32]. Unfortunately, data for “liver-up”, lung-to-head ratio, and other possible risk factors were not registered in a consistent and structured manner throughout the study period. Likewise, ventilator associated parameters such as pCO_2_ and oxygenation index (OI) were not retrievable in a consistent manner, but would have added valuable information to the study as possible indicators of severity.

Similarly, we only reported infant mortality. However, improved understanding and treatment of CDH, may result in more severely affected infants surviving, therefore it becomes relevant to evaluate post-intensive-care conditions that affect childhood morbidity and quality of life, e.g. bronchopulmonary dysplasia (BPD), requirements for tracheostomy, delayed neurodevelopment and failure to thrive [33, 34].

Our data did not include spontaneous abortion cases, terminated pregnancies due to a CDH prenatal diagnosis, or stillborn infants with CDH. Also, infants born alive and diagnosed postnatally at other hospitals, but not surviving transport to our centre, were not be included, in contrast to a similar infant born at our centre. Inclusion of these cases would have increased overall CDH mortality, an issue previously described as ‘The hidden mortality of CDH’, and discussed by other authors [1, 35]. This issue was not addressed here.

## Conclusions

We reported data on CDH survival, over an 18 year time period, using a well-defined and consistent management strategy, without ECMO. Our results were comparable with other centres, and support the need for further studies on the role of ECMO treatment for the management of CDH infants, also regarding the long-term outcomes.

## Data Availability

The datasets used or analysed during this study are available from the corresponding author on reasonable request.

## Abbreviations

CDH: Congenital diaphragmatic hernia
CMV: Conventional mechanical ventilation
HFO: High frequency oscillatory ventilation
LHR: Lung-to-head ratio
ECMO: Extracorporeal membrane oxygenation
ELSO: The Extracorporeal Life Support Organisation
CDH EURO consortium: The congenital diaphragmatic hernia European consortium.
